# The Automatic Methods Group *Newsletter*

**DOI:** 10.1155/S1463924695000307

**Published:** 1995


Journal of Automatic Chemistry, Vol. 17, No. 5 (September-October 1995), pp. 195-200

AUTOMATIC METHODS GROUP

Analytical Division
ROYAL SOCIETY OF CHEMISTRY

### The Automatic Methods Group *Newsletter*

The AMG Newsletter is published as part of and in collaboration with the *Journal of Automatic Chemistry*. Further information on the *Journal of Automatic Chemistry* can be obtained from Taylor & Francis Ltd, Rankine Road, Basingstoke, Hampshire RG24 8PR or e-mail: info@tandf.co.uk; worldwide web home page: http: ||tandf.co.uk.

### Meeting Reports

### *Automated non-invasive monitoring*

*An afternoon seminar on 'Automated non-invasive monitoring' was held by the AMG on 11 April 1995. Chaired by Professor Paul Clarke of UMIST, there were four presentations. Abstracts, for those unable to attend, follow.*

### Abstracts of papers presented

#### Quality assurance by on-line colour and infrared reflectance systems

**R. F. Edgar**
*Infrared Engineering Ltd*

In many industrial processes, considerable operating benefits can be gained by moving analysis out of the laboratory and onto the actual process stream. In-process analysis imposes very stringent demands, since the measurements may be directly or automatically acted upon without any examination or verification by analyst or competent technician. These demands lead to the need for robust measurement, in both the physical and statistical senses of 'robust'.

A number of techniques for achieving robustness were reviewed by Dr Edgar. Some of these guide initial choice of measurement principle. The acceptability of strategies for which the primary justification is statistical and correlative rather than causal and based on known chemical or physical principles was examined.

Other techniques such as standardization, linearization and ratio-based measurement represent prudent ways of either identifying and correcting for potential sources of error, or processing raw sensor data in ways in which drift, instability and other error sources may be cancelled out. These were illustrated by examples drawn from on-line experience of filter-based photometric instruments in a range of industries. These instruments include near infrared diffuse reflectance sensors for measurements such as moisture, fat and protein in foodstuffs, and on-line colour measurement.

The development of a near infrared transmittance sensor for on-line measurement of alcohol and original gravity under brewery operating conditions was also described. This case history illustrated the need to understand fully the environmental factors which any given sensing technique must be able to tolerate in the long term.

#### Open path gas measurement and calibration in environmental monitoring

**Martin J. T. Milton and P. T. Woods**
*National Physical Laboratory*

NPL has been involved in the development of new techniques for measuring gases in the atmosphere for more than 15 years. These techniques are based on the selective absorption of light by the species being measured over an open measurement path. They provide an accurate means of probing environmental air pollution issues that are often difficult to investigate using conventional instruments.

Two types of instrument developed by NPL were described:

(1) A portable monitor capable of making measurements of average concentration along a path of up to 500 m.

0142-0453/95 $10.00 © 1995 Taylor & Francis Ltd.

(2) A sophisticated Differential Absorption Lidar (DIAL) system capable of making range-resolved measurements of species with a range of up to 3 km.

These instruments have been used by NPL for a wide range of industrial measurement problems. Applications to the oil, chemical and landfill industries were described.

The calibration of optical open-path measurement systems gives rise to several technical and scientific problems. The approach taken at NPL has been to develop gas cells that are suitable to be used external to the measurement system. These have the advantage that they introduce an accurately known concentration of gas directly into the measurement path itself.

The design of a cell for use in the measurement path of a DIAL system is more complex because it must be designed to minimize any backscattering of the probe beam. This means that its diameter must be at least eight times the diameter of the probe beam and it cannot use a window to contain the gas. A cell has been constructed at NPL with a diameter of 1 m and a length of 10 m. It entrains a constant flow of the target gas into a stream of air that is sucked in through the open ends of the cell and expelled by fans around the circumference at the centre. The uniformity and accuracy of the concentration within the cell have been checked using calibrated open-path optical and point-sensing techniques. It has been used to test the performance of a DIAL system measuring methane at a wavelength of 3·4 μm.

### Acoustic emissions—a tool for chemical processing applications

**Jonathan Bouchard**
*AEA Technology*

The sound generated in a chemical process vessel can be interpreted and used to monitor the progress of a reaction taking place in the vessel. This simple, and apparently obvious, assumption underlay a significant project to apply acoustic emission monitoring techniques to the chemical process industry. Applications were identified ranging from bulk production of chemical intermediates, to batch manufacturing of pharmaceutical products. In all cases, a clear and repeatable 'noise signature' could be identified which was characteristic of the reaction process. One of the principal noise sources identified was that of crystalline product particles being struck by the vessel agitator. Trials showed that not only was it possible to trace the progress of a reaction, but that, using neural network and pattern recognition techniques, it was also possible to distinguish between suspended solid particles of different size. However, it was found that in many cases noise sources other than crystal collision dominated, and in these cases it was not possible to simply relate signal strength to particle size or concentration. Each process that was monitored was found to have its own unique characteristics.

The principal advantages of the acoustic monitoring technique were that it was relatively cheap; that a single sensor could monitor acoustic signals from throughout the volume of a vessel; that the technique was non-invasive; and that acoustic systems could easily be fitted to existing plant. The principal disadvantages were that signal interpretation was not straightforward, and that there was no simple theoretical model which could be used in all cases to link the acoustic signal to process parameters such as particle size or product yield.

### Automated non-invasive monitoring: an artificial nose—an advanced system for quality testing

**Tom Large**
*AromaScan plc*

The measurement of volatiles, odours and aromas gives a direct assessment of the sensory value and therefore quality of many products and processes. The ability to recognize and report 'trueness to type' of an aroma would therefore lend itself to a wide variety of applications involving 'yes/no', 'good/bad', decision-making in the manufacturing environment.

Traditionally, assessment of aroma is based on subjective judgement using a panel of trained personnel, or by analysis of a limited number of components in the overall aroma. Such techniques are labour intensive, expensive and prone to error. Over the past 10 years, significant advancements in the development and application of electronic aroma analysis have been achieved using conducting polymer technology. Parallel developments in microelectronics and advanced artificial intelligence in computer technology, has led to the development of electronic aroma analysers.

The need to improve final product consistency is dependent on raw material quality assurance and closely controlled process parameters. The use of novel gas sensor technology for quality assessment purposes allows for the analysis of the chemical variations in an aroma profile by the graphical presentation of data. Olfactroscopy is the term used to describe the electronic sensory analysis of aromas.

The principle of operation of the sensors is the analysis of volatile chemical species by their interaction with semi-conducting polymer sensors grouped in an array. Conducting polymers exhibit changes in electrical resistance due to adsorption and desorption of volatiles onto each sensor element. This is a rapid process at room temperature where adsorption occurs as a result of the steric, hydrophobic and ionic nature of the interactions.

This presentation described the fundamental principles of operation and data interpretation, together with an overview of the breadth of application identified thus far.

### *Soil and ground water*

*The Automatic Methods Group of the Analytical Division, Royal Society of Chemistry held a meeting on 'Soil and ground water: automatic field and laboratory testing methods' on 17*

*May 1995 at BP International's Sunbury Research and Engineering Centre. The Chairman of the morning session was Dr K. Saunders, Vice Chairman of the Group and the meeting was chaired in the afternoon by Rob Finney of BP International, Sunbury. Abstracts of some of the presentations at the meeting follow.*

### Abstracts of papers presented

### The impact of UK and EC legislation

**Mary R. Harris**
*Clayton Environmental Consultants Ltd*

The potentially adverse effect of industrial and other activities on the soil and water environment has been a matter of increasing concern in many advanced industrialized countries over recent years. In line with such concerns, the UK has developed a comprehensive regulatory framework for the protection of soil and water, of which the Environmental Protection Act (EPA) 1990 and Water Resources Act (WRA) 1991 are perhaps the most important elements. For example, the EPA 1990 covers the authorization of prescribed (industrial) processes and waste management issues, including the definition of controlled waste, waste management licensing and the Duty of Care for waste. The WRA 1991 contains the main provisions on the classification and protection of the water environment, as well as powers to prevent and remedy the pollution of controlled waters. It is through such legislation that the UK addresses European environmental protection requirements, such as the Directive on ground water protection, the Framework Directive on Waste (recently updated) and the new Directive on Hazardous Waste. An important aim of much of the legislation is to minimize, or prevent, soil or water pollution, although remedies are available where breaches of the relevant controls take place. The common law also provides an important potential mechanism for action where the polluting activities of one party adversely affects the interests of another.

The existing statutory framework, together with common law provisions, is considered sufficient both to deter behaviour/activity likely to lead to environmental damage and as a means of redress in the event that damage occurs. However, draft legislation (the Environment Bill) currently before the UK Parliament contains provisions that will mark two important changes in the current regime. The first is the creation of two new unified environment protection agencies (one for England and Wales and one for Scotland), which will inherit the control functions of the existing regulatory bodies. The second concerns the specific responsibilities of both local authorities and the environmental agencies in relation to land which complies with a new statutory definition of contaminated land.

Dr Harris summarized the main current and proposed legislative provisions on soil and water protection, and provided examples of the important technical role that monitoring could play in ensuring that legislative requirements are met.

### Qualitative assurance for soil and ground water analysis

**James W. Farrell-Jones**
*Geochem Group Ltd*

The analysis of soil and ground water has become a major new area of environmental chemistry over the last 10 years. The data generated is employed to assess the degree of environmental risk and therefore to assist decision-making with regard to property transactions and remediation options. Such decisions have major financial and legal implications for property developers, industrial operators, insurance underwriters and financial backers who need to be confident that they are making their decisions from a position of sound knowledge. How can these groups be certain that the available information describing the condition of a site is accurate and can be relied upon?

Site investigation and remediation contracts can generate large numbers of samples for analysis in comparatively short periods of time, and laboratories which deal with samples of this type are increasingly required to process samples quickly. Volume and speed are attributes of the market that can be addressed in practical ways simply by ensuring that the laboratory is properly equipped and adequately staffed, but quality is often taken for granted both by users of the data and the laboratories themselves.

There are many variables to be considered in assessing soil and ground water contamination, and considerable attention has been paid to sampling and the errors that this can introduce. However, much less attention has been given to the variability that results from matrix effects, sample preparation techniques, extraction and digestion procedures and instrument bias. The quality of the final product, by which we mean the validity of the data, depends upon the analyst being able to quantify the effects of these sources of error. Although it has often been said that sampling errors significantly outweigh analytical errors, this should not be an excuse for complacency in the laboratory or on the part of the interpreter. Environmental analysis is about being able to detect very small amounts of diverse substances—regulatory standards are often set close to the limits of detection and at these levels, small errors can make major differences.

This presentation described how a NAMAS Accredited commercial laboratory ensures that the data it generates is valid. An example of a routine analysis for petroleum hydrocarbons was used to illustrate how difficult this is to achieve and the procedures that can be put in place to overcome these problems.

### Field screening of contaminated soil and water

**R. W. Finney**
*BP International Research and Engineering Centre*

Surveys of potentially polluted sites in advance of remediation, acquisition and divestment can be expensive and protracted, with measurement and characterization of pollutants being a major financial burden. Use of field portable analysis can provide a speedy and cost-effective means to identify major analytes, to delineate the extent of the contamination and to locate 'hot-spots' for immediate installation of boreholes. The capability to obtain analytical data in the field and to act on them, results in a focused survey where trends can be followed and ensures sampling for laboratory analysis is carried out to maximum effect. Field screening for general organics can comprise the use of a portable PID, FID or pellister/thermal conductivity based analyser or the field test kit approach. Two test kit technologies are applied for TPH determination, Friedel-Crafts alkylation and immunoassay. The former serves to produce a precipitate after reaction of extracted aromatics with an alkyl halide in solution. The colour of the precipitate can be characteristic of the nature and provide semi-quantitative estimates of the organic material. Quoted detection limits from water and soil are 0·10 and 1·00 ppm respectively. Immunoassay kits use a competitive interaction between the extracted target analyte(s) and an enzyme conjugate for an antibody raised against the analyte suite (usually BTEX). Developed colour can be compared with a colour chart or preferably read using a photometer. Quoted detection limits are sub-ppm for water and 2·5–10·0 ppm for soils. In some cases, a result confirming absence/presence of analyte against a pre-defined target limit is obtained. Some cross-reactivity can occur, for example, the BTEX kit reacts with PAHs, so measurements should be considered semi-quantitative. Workstation design aids convenient usage and negates special disposal requirements.

The cost can be as low as £10 per sample for batches and, generally, results are available within 10–15 min. Although good correlations have been obtained with approved laboratory methods, field testing using portable analysers and test kits should not be seen as a replacement for these but as a means to target where most effectively to sample for laboratory testing and to select locations where laboratory analysis would be most productive.

### Detailed analysis of organic pollutants and breakdown products in contaminated soil and ground water

**Robert Large**
*M-Scan Ltd*

Contaminated site surveys normally incorporate routine screening of organic pollutants using such parameters as total extractables, total petroleum hydrocarbons (TPH), BTEX, etc. While such parameters are necessary and cost-effective for estimating and mapping the distribution of organic pollution on the site in question, there is also a need for detailed organic analysis on a selective basis.

When detailed analysis *is* included in a site survey, it is normally based on the target pollutant approach, since this can be easily automated in a GC/MS instrument. This approach allows defined (toxic) pollutants to be determined specifically, an important requirement. There is a risk, however, that the information obtained defines what is *not* present rather than what *is* present.

This limitation can be overcome by the combination of appropriate methods (GC/FID, GC/ECD, GC/EIMS, GC/CIMS, GC/HRMS, FABMS, LC/MS, probe EIMS, DCI etc.) and detailed interpretive expertise. This 'problem-solving' approach can allow:

(1) Detailed characterization, speciation and ageing of refined product inputs.
(2) Characterization and determination of biodegradation and photodegradation intermediates and metabolites.
(3) Characterization of unusual/unexpected organic pollutants, particularly where library mass spectra are not available.
(4) Measurement of the efficacy of remediation treatments.
(5) Determination of the fate of organic pollutants.

The approach used by M-Scan was illustrated in the presentation by reference to specific case studies.

### Monitoring of river and ground water

**Mark Kibblewhite**
*Acer Environmental Laboratories and Sciences*

EC Directives require organic pollutants in drinking water to be monitored at below 0·1 ppb. The analytical performance should provide a detection limit of at least 0·01 ppb and a total measurement error of better than 20%. In the UK, the National Rivers Authority has the statutory responsibility for monitoring river and ground waters used for drinking water abstraction. Water companies have no statutory need to monitor the quality of the raw water they abstract for treatment (but they must monitor the quality of water they supply). However, there are powerful operational reasons for monitoring the levels of organics in raw waters—which can be simply summarized as 'protection of supply'. Knowledge that raw water is not within specification in respect of organic pollutants allows prompt responses such as closure of intakes, pumping from storage, switching to alternative supplies, and, in extreme cases, ceasing supply to avoid pollution of the distribution network.

Organic pollutants can be expected or unexpected. Pesticide use in a river catchment and past experience may inform a need to monitor for specific compounds. Alternatively, where a catchment contains a wide range of industrial activities, there should, ideally, be general background monitoring of organic pollution to provide early warning in the event of spills or unauthorized discharges. The use of on-line monitors for both predictable and unpredictable pollutants has many advantages because it offers continuous real time data, but the approaches needed for these two scenarios are different.

The presence of pesticides from normal agricultural operations is common in surface waters; the loading varies seasonally, reflecting the annual cycle of crop husbandry. When combined with rainfall variability, there is the potential for exceeding the limits, even where levels are not normally a problem. Present practice is to increase the frequency of sampling and laboratory-based analyses at times of higher risk. But such an approach provides long response times for sampling-to-results and less than robust protection of supplies. But the need for operational robustness and high analytical sensitivity means that the development of effective on-line monitoring of this kind is challenging.

Measurement of uron pesticides is an example of a current requirement for compound specific monitoring. Established practice in Acer's laboratories is to measure these by a combination of solid phase extraction (SPE) and high performance liquid chromatography (HPLC), using an initial sample volume of 2 l. An automated system named SAMOS (System for the Automatic Measurement of Organic micro pollutants in Surface water) is presently being assessed. It consists of an HP1090 Win liquid chromatograph combined with a diode array detector. SPE is performed on a programmable solid phase extraction module (PROSPEKT), combined with a solvent delivery unit. Up to 100 ml of water is pumped into a disposable cartridge with a 15 to 25 µm reverse phase material, and subsequently the urons are gradient eluted from the cartridge to the chromatograph. Results obtained using SAMOS compare favourably with those using normal procedures. Limits of detection are very similar, while SAMOS provides improved precision. The advantages of a smaller sample volume and the complete sample-to-result automation offered by SAMOS opens the way for us to introduce *in situ* on-line analysis of these pesticides.

A different approach is needed where the nature of the organic pollution cannot be pre-determined. In theory, a range of compound-specific on-line analysers could be developed to provide a sufficient scope of analytes; for example, SAMOS could be combined with an automated GC-MS system and a total organic carbon analyser. In practice this approach is too sophisticated and expensive for what is really an alarm problem—because true quantitation is not needed. Acer are currently investigating the potential use of non-specific analysis to provide this alarm function. In essence, the objective is to deploy a system which is capable of producing and analysing sets of physical response data and recognizing that the state of the raw water has changed from an expected set of conditions. A number of commercial devices have been developed which predict basic parameters such as solids contents and total organics loadings by the multiple regression analysis of data from pH, conductivity, turbidity, and UV–visible absorbance sensors. The next logical step is to analyse outputs of this type using pattern recognition techniques, such as principal components analysis, so that dimensions can be established for 'normal' raw waters and those which contain evidence of organic pollutants such as hydrocarbons, chlorinated solvents and phenols.

### Field analytical technologies

**Dale L. Bacon**
*3M Environmental Laboratory*

Set within the context of the complex environmental analytical needs of a multinational manufacturing company, Dale Bacon presented the 'real world' strategies and tactics employed by 3M Environmental Laboratory to utilize state-of-the-art field analytical technologies to help achieve corporate goals. The presentation discussed the integral role played by field analytical technologies within the structure of a full service environmental laboratory. It profiled the technologies and capabilities of the 3M Field Analytical Technologies Group and featured several illustrative case studies including: site remediation support, soil gas and metal analyses, and emissions monitoring.

### Water analysis in the petrochemical industry

**M. H. Henderson**
*BP International, Research and Engineering Centre*

It is BP's policy to focus on activities that keep BP in business. The Health, Safety and Environmental (HSE) Shared Resource provides an expert resource and consultancy to BP businesses for ambient air, environmental analysis, fugitive emissions, occupational hygiene, safety, fire and risk management, safety physics, and soil, effluent and water activities.

The approach of the HSE Shared Resource to water analysis was illustrated in this presentation with reference to case histories requiring a diverse range of wet chemical and advanced spectroscopic techniques available at Sunbury. Petroleum contamination, heavy metal contaminants in effluents and Volatile Organic Compound (VOC) emissions from water treatment facilities were discussed. Generally, a range of complementary techniques is required to reach a satisfactory conclusion. The factors involved in obtaining a suitable sample for analysis were discussed.

The HSE Shared Resource evaluates the suitability of specific analyses for BP, and compares the performance of different methods to screen for the same analyte. Technical differences in the methods specified by the US EPA, BSI, DIN, NEN, etc., for selected analytes were discussed.

### Forthcoming Meetings

### Royal Society of Chemistry & Royal Pharmaceutical Society of GB

### A joint meeting of The Automatic Methods Group & The Joint Pharmaceutical Analysis Group on Thursday 7 December 1995, at the Royal Pharmaceutical Society of GB, 1 Lambeth High Street, London SE1 7JN

*Robotics and Automation in the Pharmaceutical Laboratory*

### Programme

| | |
|---|---|
| 10.00 | Registration |
| 10.30 | Introduction & speculation<br>Ken Leiper, Glaxo Pharmaceuticals |
| 11.05 | State of the art in analytical robotics<br>Prof Kevin Warwick, University of Reading |
| 11.40 | Microbiological assays<br>Ken Coleman, SmithKline Beecham |

| | | |
|---|---|---|
| 12.15 | On-line NIR<br>Ron Belchamber, Process Analysis and Automation |
| 12.50 | Lunch |
| 14.00 | Water analysis<br>Robin Andrew, NW Water |
| 14.35 | Automation in pesticide analysis<br>Keith Parsley, MAFF, Norwich |
| 15.10 | Automated applications in bioanalysis<br>Gordon Plummer, Zeneca Pharmaceuticals |
| 15.45 | A self-service laboratory<br>Dr Don Clark, Pfizer Research, Sandwich |
| 16.20 | Close of meeting |

See Forthcoming AMG Programme for further information contact.

### Monitoring for the Needs of Society: New Horizons in Pollution Control

**A joint meeting of the Automatic Methods Group and the South East Region of the Analytical Division of the Royal Society of Chemistry in co-operation with the Health & Safety Executive. Tuesday 12th and Wednesday 13th December 1995, at the Scientific Societies Lecture Theatre, New Burlington Place, London W1**

The symposium brings together a large number of experts in the field to explain recent developments in measurement and testing used for assessing the quality of life. The meeting is designed to examine the relationship between legislation, standards and current practice with a view to closer harmonization of best practice and to identify practical difficulties in meeting current and likely future legislation. The needs for further developments, particularly in direct and automated measurement, will be identified and the areas where there can be harmonization of methodologies between indoor, ambient and workplace monitoring highlighted.

The meeting will be of interest to environmental practitioners, safety engineers, occupational hygienists and industrialists wishing to become familiar with new monitoring and analysis technologies and to learn from the experience of others.

**Programme**

The first session will focus on the application of *Directives and Standards* to workplace, indoor air and environmental monitoring. The second session, entitled *The Framework for Measurement*, will look at quality assurance issues with papers on accreditation as well as calibration and traceability. The third session will be a wide-ranging examination of all aspects of *Measurement.* There will be papers on thermal-desorption techniques, hand-held instruments, and detector tubes. Part of this session will also be devoted to several papers on different monitoring strategies. The fourth, and final, session, entitled *New Horizons for Pollution Control*, will look at new measuring techniques and will include papers on a piezoelectric ozone monitor, the Electret dust sampler, a portable monitor for asbestos, the 'electronic nose' and a portable XRF spectrometer.

Speakers from the UK, USA, Sweden, The Netherlands, Germany and Italy are drawn from the ranks of regulatory authorities and laboratories, local authorities, research establishments, universities, instrument manufacturers and consultants.

The symposium will be supported by a poster session on both days. Potential delegates are invited to submit posters on subjects related to the theme of the symposium. A prize of £100 will be presented for the best poster and £50 to the runner-up.
See Forthcoming AMG Programme for further information contact.

### Forthcoming AMG Programme

| Date | Title | Venue |
|---|---|---|
| 7 Dec 1995 | Robotics and Automation in the Pharmaceutical Laboratory<br>(Held in conjunction with the Joint Pharmaceutical Analysis Group) | London |
| 12–13 Dec 1995 | Environmental Monitoring III: Monitoring for the Needs of Society: New Horizons<br>(Held in conjunction with the South East Region of the AD) | London |
| 28–29 Feb 1996 | LIMS Conference<br>(Held in conjunction with the Flemish Chemical Society | Kortrijk, Belgium |
| Spring 1996 | The Industrial/Academic Interface<br>(Held in conjunction with the Western Region AD) | Bristol |
| May 1996 | Automated Process Monitoring (BP-IV) | BP Hull |
| Nov 1996 | Automation & Laboratory Analysis | London |
| Dec 1996 | Environmental Monitoring IV: Field Test Kits: Clinical & Industrial Approaches Compared | London |
| 1996 | Analytical Uncertainty | London |
| 1997 | Laboratory Automation and Pharmaceutical Production | UK |
| May 1997 | BP-V | UK |
| Dec 1997 | Environmental Monitoring V | UK |
| 11–13 Jun 1997 | 11th International LIMS Conference | UK |
| Dec 1997 | Environmental Monitoring V | UK |
| 1998 | Health & Safety at Work | Luxembourg |

**Further information:** For further information about any of the meetings listed above, or if you wish to be placed on our mailing list, please send your name and address to: Dr R Narayanaswamy, DIAS, UMIST, PO Box 88, Manchester, M60 1QD. Tel: 0161 200 4891/4885: fax: 0161 200 4911. **Poster session:** If you wish to submit a poster for the Environmental Monitoring meeting on 12/13th December please send an abstract of approximately 100 words to Dr Narayanaswamy by 30th October 1995.

